# Learning Hierarchically Consistent Disentanglement with Multi-Channel Augmentation for Public Security-Oriented Sketch Person Re-Identification

**DOI:** 10.3390/s25196155

**Published:** 2025-10-04

**Authors:** Yu Ye, Zhihong Sun, Jun Chen

**Affiliations:** 1 National Engineering Research Center for Multimedia Software, School of Computer Science, Wuhan University, Wuhan 430072, China; ms.yeyu@whu.edu.cn; 2 Hubei Key Laboratory of Multimedia and Network Communication Engineering, Wuhan University, Wuhan 430072, China; 3 Department of Information Security, Naval Engineering University, Wuhan 430032, China; zhihong.sun@whu.edu.cn

**Keywords:** sketch re-identification(Re-ID), cross-modality, feature disentanglement, public security

## Abstract

Sketch re-identification (Re-ID) aims to retrieve pedestrian photographs in the gallery dataset by a query sketch image drawn by professionals, which is crucial for criminal investigations and missing person searches in the field of public security. The main challenge of this task lies in bridging the significant modality gap between sketches and photos while extracting discriminative modality-invariant features. However, information asymmetry between sketches and RGB photographs, particularly the differences in color information, severely interferes with cross-modal matching processes. To address this challenge, we propose a novel network architecture that integrates multi-channel augmentation with hierarchically consistent disentanglement learning. Specifically, a multi-channel augmentation module is developed to mitigate the interference of color bias in cross-modal matching. Furthermore, a modality-disentangled prototype(MDP) module is introduced to decompose pedestrian representations at the feature level into modality-invariant structural prototypes and modality-specific appearance prototypes. Additionally, a cross-layer decoupling consistency constraint is designed to ensure the semantic coherence of disentangled prototypes across different network layers and to improve the stability of the whole decoupling process. Extensive experimental results on two public datasets demonstrate the superiority of our proposed approach over state-of-the-art methods.

## 1. Introduction

With the rapid advancements of emerging information technologies such as artificial intelligence and the Internet of Things [1,2,3], urban public safety has become one of the foremost concerns in daily life. As a key supporting technology in intelligent video surveillance systems within the public security domain, person re-identification (Re-ID) is a fundamental task in intelligent video surveillance systems that match a known person from a large photo dataset captured by disjointed cameras using a query image [4,5]. This technology demonstrates broad applicability in urban public safety initiatives and commercial applications. However, query images are not always available in practice due to surveillance blind spots where monitoring equipment fails to provide sufficient coverage. In such circumstances, only eyewitness accounts may be available, while photo evidence of suspects remains absent. To this end, sketch person re-identification (Sketch Re-ID) has been proposed that utilizes eyewitness descriptions to draw professional sketches as queries and to match target photos in the gallery database, has received widespread attention from researchers and scholars.

**Figure 1:**
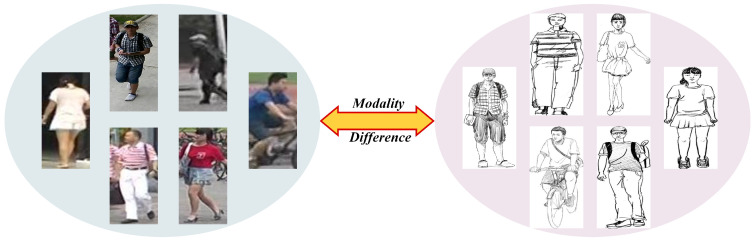
Cross-modal correspondences between photo and sketch images.

Matching sketches with photographic images is a highly challenging task due to the large modality gap. As depicted in Figure 1, this discrepancy arises from fundamental differences in their imaging principles, which leads to heterogeneity and information asymmetry between the two modalities. For instance, sketches are highly abstract, capturing only the structural and contour information of a subject while completely lacking the rich color and texture details present in photos. To overcome this, a popular strategy is to learn a generic latent feature embedding space for sketches and photos via cross-modal metric learning, then achieve hard alignment. However, due to the inherent information asymmetry, such hard alignment strategies often fail to effectively capture the complex dependencies and correlations across modalities.

An alternative approach introduces intermediate modalities to bridge the two source modalities. For example, Chen [6] generates images from various spectral combinations, which facilitates learning cross-modal invariant features. Recent works [7,8] employ an asymmetric disentangled learning approach to decompose photographic features into sketch-related and sketch-unrelated components, and then use generative adversarial networks to synthesize sketch images, thereby reducing the modality gap between sketches and photographs. However, these methods perform feature disentanglement only at a single modality and a single scale. Moreover, inherent limitations in generative performance introduce noise and artifacts that compromise the precision of cross-modal matching.

To address these limitations, we propose a novel multi-channel augmentation with hierarchical disentanglement method that mitigates modality gaps at both the data and feature levels. At the data level, to mitigate the color-information asymmetry between sketches and photos, our method employs multiple color augmentation strategies to reduce the reliance on color cues and encourage the model to extract more robust modality-invariant representations, such as structural and contour features. At the feature level, we introduce a modality-disentangled prototype(MDP) module that decomposes pedestrian representations into modality-invariant structural prototypes and modality-specific appearance prototypes. Unlike prior works [7,8] that only perform single-layer disentanglement on the photo modality, our MDP modules explicitly decompose pedestrian representations on both sketch and photo modalities across different network depths. Furthermore, to ensure semantic coherence of disentangled prototypes across different network hierarchies, we incorporate a cross-layer decoupling consistency constraint, which significantly enhances the stability of the decoupling process. In summary, the main contributions of this paper are summarized as follows:

(1) A multi-channel color augmentation(MCCA) module is proposed to address the information asymmetry between sketch and photo modalities. By applying diverse color transformations to the input images, the network is encouraged to discover modality-invariant features beyond color cues.
(2) A modality-disentangled prototype(MDP) module is introduced to disentangle sketch and photo features into structural and appearance prototypes at different network layers. Moreover, a cross-layer decoupling consistency constraint is incorporated to ensure that the decoupled representations maintain coherent semantic information across different network hierarchies.
(3) Experimental results on the public sketch Re-ID dataset demonstrate the effectiveness and superiority of our proposed method through comprehensive experiments against state-of-the-art (SOTA) approaches.

The remainder of the paper is organized as follows. Section 2 reviews related work on cross-modal person re-identification, sketch–photo person re-identification, and disentangled representation learning. Section 3 introduces our multi-channel augmentation-based hierarchical disentanglement network framework. This section elaborates on the Multi-Channel Color Augmentation (MCCA) module, the Modality-Disentangled Prototype (MDP) module, and the proposed cross-layer decoupling consistency constraint. Section 4 describes the experimental setup and reports comprehensive evaluation results to validate the effectiveness of our method. Finally, Section 5 concludes the paper and outlines directions for future work.

## 2. Relate Work

### 2.1. Cross-Modal Person Re-Identification

With the rapid growth in urban public safety requirements, single modal person re-identification methods have become inadequate for practical applications. This limitation arises because obtaining high-quality images of target individuals is often difficult in real-world scenarios. Cross-modal person Re-ID aims to match person information from different modalities. Currently, there are four main kinds of cross-modal Re-ID, including infrared-RGB ReID [9,10,11,12,13,14,15], depth image-RGB ReID [16,17,18], text-RGB image [19,20,21], and sketch-image ReID [7,8,22,23,24,25]. The main challenge faced in cross-modal person Re-ID is the substantial modality disparity between the two modalities [26]. The modal discrepancies addressed by these cross-modal recognition methods vary, due to differences in imaging principles and acquired information. Consequently, directly transferring methods originally developed for other types of cross-modal matching to sketch-image matching does not yield satisfactory performance. Existing cross-modal Re-ID methods can be broadly divided into two categories: modality-shared feature learning and generative adversarial network-based learning. Modality-shared feature learning method seeks to project shared features from two modalities into a unified feature space through cross-modal metric learning, thereby obtain discriminative information for matching. However, due to significant domain gap, some modality-specific information increases intra-class variations during the feature matching process, compromising robustness to modality variations. These methods based on generative adversarial networks to generate style-aligned images and then replace the original modality. Nevertheless, such approaches inevitably introduce generation noise and require a large number of parameters for effective training.

### 2.2. Sketch Re-Identification

Sketch re-identification (Re-ID) is a critical technology in video surveillance systems that enables the identification of query pedestrians across multiple non-overlapping cameras using professional drawn sketches. As a cross-modal matching problem, the main challenge of sketch person Re-ID is mitigating the modality gap between sketch and RGB image domains. Pang et al. [22] introduced the first sketch dataset for sketch Re-ID, which uses full-body professional sketches as queries, and proposed a cross-modal generative adversarial network to extract modality-shared features. Gui et al. [23] developed a multi-level feature representation that integrates sketch and photographic modalities and used gradient reversal layers to reduce domain disparity. Yang et al. [27] applied domain adaptation techniques to transfer labels from other datasets to the sketch domain. Chen et al. Several studies applied attention mechanisms [28] to learn cross-modal invariant features. Zhu et al. [24] designed a cross-domain attention mechanism to capture identity-related features and their cross-modal relationships, and they proposed a cross-domain center loss to draw features from the two modalities closer together in feature space. Refs. [6,29] introduced a multi-spectral fusion strategy by generating images from various spectral combinations (e.g., grayscale images, single-channel representations), which helps learning cross-modal invariant features. However, this approach aligns modalities at the data-preprocessing stage and does not explicitly reduce feature-distribution differences in the embedding space. Lin et al. [30] delivered a large-scale sketch dataset named Market-Sketch-1K and proposed a dual-stream network with non-local attention to learn modality-invariant features. However, this method overlooks the significant interference in cross-modal matching caused by the inherent color information asymmetry. Chen et al. [7,8] employed a generative adversarial sketch synthesis network to generate auxiliary sketch modalities, thereby increasing the diversity of training samples. However, auxiliary modalities inevitably introduces noise into the training process. In this paper, we introduce a multi-channel augmentation module for sketch-photo matching does not rely on auxiliary generative networks. In this paper, we introduce a multi-channel augmentation module for sketch–photo matching that does not rely on auxiliary generative networks. The proposed method expands the training set by systematically applying diverse color transformations to input images, thereby reducing the interference caused by inherent color-information asymmetry across modalities.

### 2.3. Disentangled Representation Learning

Disentangled representation learning seeks to decompose entangled object representations into independent and semantically interpretable factors and to improve the model’s applicability to real-world scenarios [31]. Common disentangled representation paradigms include variational autoencoders (VAEs), generative adversarial networks (GANs), and cross-regularization methodologies. Gatys et al. [32] introduced a content–style disentanglement paradigm that encoded modality-invariant features as content while encoding domain-specific features as style. Lee et al. [33] decomposed an image into a domain-invariant content space and a style space capturing domain-specific attributes, enabling diverse style transfer between unpaired images. Building upon disentanglement principles, Qian et al. [34] decomposed pedestrian images into clothing and shape information to address cloth-changing Re-ID challenges. Sain et al. [35] proposed a disentanglement model that separated sketch and photographic features into content-related and style-related representations. Similarly, Chen et al. [7,8] applied disentanglement concepts to decompose photos into sketch-related and sketch-irrelevant factors, and they subsequently employed auxiliary generated sketches to transfer sketch-irrelevant factors to the sketch modality.

Inspired by disentangled learning, our proposed method simultaneously disentangles features into cross-modal invariant structural prototypes and modality-specific appearance prototypes. Unlike prior work, we emphasize the discriminative power of disentangled features for cross-modal recognition rather than generating cross-domain images for alignment. Furthermore, we first apply disentanglement to both sketch and photo features across different network depths, and we introduce a cross-layer decoupling consistency constraint to improve the stability of the whole decoupling process. Experimental results demonstrate the superior efficacy of our approach for sketch–photo cross-modal matching.

## 3. Method

In this section, the whole framework of our proposed method will be introduced first, followed by the multi-channel color augmentation module, the modality-disentangled prototype module and cross-layer decoupling consistency constraint will be described in detail.

### 3.1. The over All Framework

**Figure 2:**
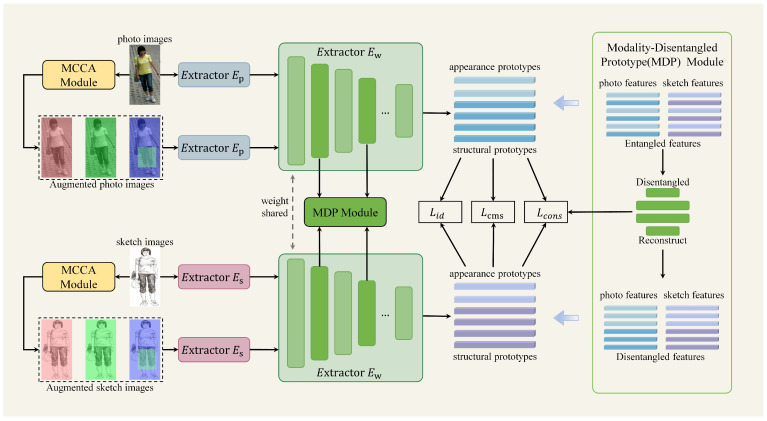
The framework of the proposed method.

In this section, we describe the details of our multi-channel augmentation-based hierarchical disentanglement network framework. As shown in Figure 2, the proposed network consists of three components, multi-channel color augmentation(MCCA) module, dual-stream feature extraction network, and modality-disentangled prototype(MDP) module with cross-layer decoupling consistency constraint loss. Suppose $V={\left\{v_{i},l_{i}\right\}}_{i=1}^{N_{V}}$ and $S={\left\{s_{i},l_{i}\right\}}_{i=1}^{N_{S}}$ represent photo images and sketch images and their corresponding labels, respectively. $N_{t}$ is the number of images, where $t\in \left(S,V\right)$. Specifically, to eliminate interference caused by color information, the input images are first processed through the multi-channel color augmentation module. This module executes diverse channel-level combinatorial transformations to simulate variations in brightness, stroke pressure, and scanning contrast between photo and sketch modalities. Then, both augmented and original images are fed into the dual-stream feature extraction network. This feature extraction network incorporates two parallel shallow branches with identical architectures, designed to capture photo features and sketch features, respectively. Additionally, a shared deep network extracts high-level semantic features. Finally, the extracted semantic features are decomposed into modality-invariant structural prototypes and modality-specific appearance prototypes by modality-disentangled prototype module. To enforce alignment across both modalities and network depths, a cross-layer decoupling consistency constraint is applied to prototypes of the same semantic class at different network stages.

### 3.2. Multi-Channel Color Augmentation Module

Photographs captured by visible-light cameras contain rich color information, whereas sketch images lack color and typically appear as monochrome images characterized by variations in line thickness and shading. These inherent heterogeneity and pronounced color asymmetry across modalities significantly degrade cross-modal recognition performance. The core idea of our proposed multi-channel color augmentation(MCCA) module is to generate a diverse set of augmented images that simulate the stylistic variations between photos and sketches, such as differences in brightness, stroke pressure, and scanning contrast. By reducing the model’s reliance on color cues, our multi-channel color augmentation module encourages learning of more robust, modality-invariant features such as structural outlines and contours.

**Figure 3:**
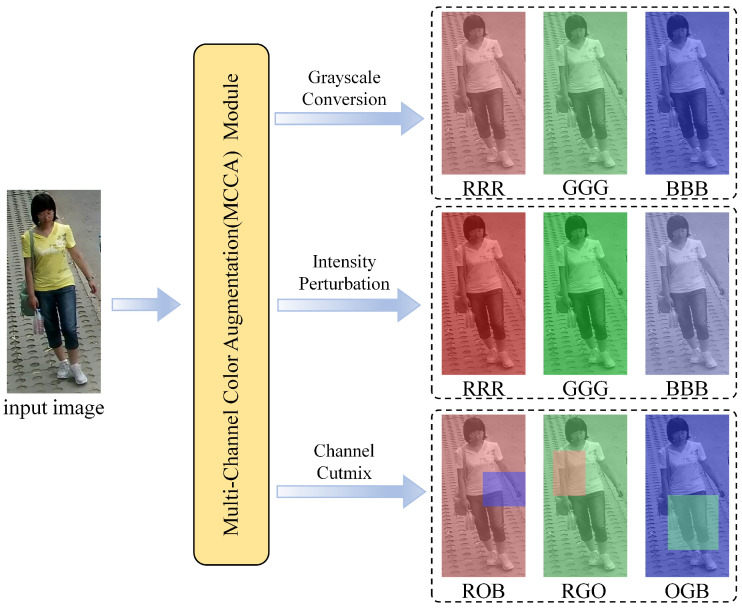
Illustration of the proposed multi-channel color augmentation module. R/G/B/O represent R channel, G channel, B channel, zero padding, respectively.

Figure 3 illustrated the details of our MCCA module. We take photographic images as example to demonstrate the augmentation process. For each input image *V*, our MCCA module employs three augmentation strategies:

1. Grayscale Conversion: The image is converted to a grayscale representation. This is achieved either by randomly selecting one of the R, G, or B channels and replicating it three times, or by applying a standard weighted-sum conversion. The resulting grayscale image is denoted as $\hat{V}$.

2. Intensity Perturbation: Building upon the first strategy, a random intensity shift is introduced to the grayscale image $\hat{V}$. This process is formulated as:

(1) $$I=\alpha \cdot \hat{V},$$

where α is a random perturbation factor uniformly sampled from the range [0.5,1.5].

3. Channel CutMix: This strategy first creates a two-channel representation by randomly selecting two of the three color channels (e.g., R and G) and zero-padding the third (e.g., creating an “RGO” image). Subsequently, a CutMix-style operation is performed between these two active channels, where a random rectangular patch is cut from a source channel and pasted onto the corresponding location in the target channel. This can be expressed as:

(2) $$I=\mathrm{CutMix}(C_{\mathrm{src}},C_{\mathrm{tar}}),$$

where $C_{\mathrm{src}}$ and $C_{\mathrm{tar}}$ are distinct channels randomly selected from R,G,B, and the unselected channel is masked to zero.

### 3.3. Modality-Disentangled Prototype Module

Although a pedestrian’s appearance varies markedly between photo and sketch modalities, the underlying structural configuration remains invariant. To encourage the model to capture this modality-invariant structure, we introduce the modality-disentangled prototype(MDP) module. The MDP module employs spatial-prototype attention to explicitly decompose image features into structural prototypes that encode cross-modal invariant geometry and appearance prototypes that retain modality-specific cues, followed by channel-wise gating mechanism for discriminative features reconstruction. Formally, given an input feature map $x\in R^{B\times C\times H\times W}$,where *B* represents the batch size, *C* is the number of channels, and *H* and *W* represent the spatial dimensions, we first apply a 1×1 convolutional layer to project it into a two-channel attention map $M\in R^{B\times 2\times H\times W}$. These two channels are respectively designated to learn the spatial attention for the structural and appearance prototypes. Subsequently, a softmax function is applied across the spatial dimensions of each channel to obtain normalized attention weights, denoted as $M^{\prime }$, this operation can be expressed as below:

(3) $$M_{i}^{\prime }=Softmax\left(M_{i}\right),i\in \left\{0,1\right\},$$

where $M_{0}$ and $M_{1}$, after being flattened, correspond to the spatial attention weights for structural and appearance prototype attention maps, respectively.

Next, we derive the structural prototype $p_{s}\in R^{B\times C}$ and appearance prototype $p_{a}\in R^{B\times C}$ by computing a weighted average of the input features across all spatial locations, guided by the attention maps $M^{\prime }$. These operations can be formulated as:

(4) $$p_{s}=\sum_{h,w}M_{0}^{\prime }\left(h,w\right)\cdot x\left(h,w\right),\;p_{a}=\sum_{h,w}M_{1}^{\prime }\left(h,w\right)\cdot x\left(h,w\right),$$

where $x\left(h,w\right)\in R^{C}$ is the feature vector at spatial position (h,w), $M_{0}^{\prime }\left(h,w\right)$ and $M_{1}^{\prime }\left(h,w\right)$ are their corresponding scalar attention weights.

Then a channel-wise gating mechanism is employed to adaptively fuse information from the structural and appearance prototypes. Specifically, these two prototypes are stacked into a unified prototype matrix of shape $R^{B\times 2\times C}$, and a depthwise 1D convolution with kernel size 2 and group number *C* is applied to learn channel-specific fusion weights independently for different channels across the two prototypes. The result is passed through a Batch Normalization layer and a sigmoid function to generate the final gating signal, this operation can be expressed as:

(5) $$g=\sigma \left(BN\left(DWCon1D\left(stack\left(p_{s},p_{a}\right)\right)\right)\right)$$

The gating signals are then employed to reconstruct the input features *x*. To ensure training stability and promote effective module learning, we design a specialized residual connection structure as follows:

(6) $$x_{out}=x⊕BN_{zero\_init}\left(x⊗g\right),$$

where ⊕ represents element-wise addition, ⊗ represents channel-wise multiplication, and $BN_{zero\_init}$ has its learnable affine parameters $\left(\gamma ,\beta \right)$ initialized to zero. This ensures the module acts as an identity function at the beginning of training, significantly enhancing stability.

The MDP module facilitates the network’s ability to extract cross-modal invariant structural features while simultaneously enhancing feature representation capacity through gated reconstruction operations. This dual functionality enables more effective cross-modal matching by emphasizing structural consistency while accommodating modality-specific variations.

### 3.4. Cross-Layer Decoupling Consistency Constraint

We embed MDP module at multiple stages of the backbone network to perform effective feature disentanglement across different semantic hierarchies. To ensure that the disentanglement strategy remains stable and coherent as features are refined from lower to higher levels of abstraction, we introduce a novel cross-layer decoupling consistency loss $L_{\mathrm{cons}}$.

The core principle behind this constraint is that a robust disentanglement model should maintain globally consistent discriminative criteria. Specifically, the cross-layer decoupling consistency loss focuses on the network’s importance judgments regarding structural prototypes and appearance prototypes across different hierarchies. We assume that the network deems structural information more critical than appearance cues at one layer, it should maintain a similar preference at adjacent layers.

During the training process, we collect the structure prototypes and appearance prototypes from all layers into two lists for calculating the cross-layer decoupling consistency loss. To quantify the preference of structural versus appearance information at a given layer, we calculate the L2 norm of each prototype vectors and take their difference. For prototypes extracted at the l-th layer, the preference score is defined as:

(7) $$\Delta_{l}\left(x\right)={∥p_{s}^{l}\left(x\right)∥}_{2}-{∥p_{a}^{l}\left(x\right)∥}_{2},$$

where ${∥\cdot ∥}_{2}$ is the L2 norm of the prototype vector for each sample in the batch. A positive value $\Delta_{l}\left(x\right)>0$ indicates that the network at this layer emphasizes structural information, whereas $\Delta_{l}\left(x\right)<0$ suggests greater emphasis on appearance information.

The cross-layer decoupling consistency loss enforces hierarchical consistency by minimizing the absolute difference in preference between adjacent layers (e.g., layer *l* and layer l+1). This ensures that the relative importance of structure and appearance remains consistent as network depth increases. Suppose we have *L* decoupling layers, The total cross-layer decoupling consistency loss is formulated as:

(8) $$L\mathrm{cons}=\frac{1}{L-1}\sum_{l=1}^{L-1}E_{x∼B}\left[\left|\Delta_{l}\left(x\right)-\Delta_{l+1}\left(x\right)\right|\right],$$

where *L* represents the total number of stages where MDP modules are embedded, and Ex∼B[·] denotes the average over samples in the mini-batch. By minimizing Lcons, we enforce $|\Delta_{l}\left(x\right)-\Delta_{l+1}\left(x\right)|\to 0$, which encourages:

(9) $${∥p_{s}^{l}\left(x\right)∥}_{2}-{∥p_{a}^{l}\left(x\right)∥}_{2}\approx {∥p_{s}^{l+1}\left(x\right)∥}_{2}-{∥p_{a}^{l+1}\left(x\right)∥}_{2}.$$

In other words, if layer *l* prefers structural, then Lcons encourages layer l+1 to maintain a similar preference for structure, and vice versa. The cross-layer decoupling consistency loss imposes a global constraint on the entire disentanglement process, thereby enhancing the robustness of the final learned representation.

### 3.5. Overall Loss Function

During training, our proposed model is optimized via a composite loss function designed to learn discriminative features. This objective combines three distinct components: an identity loss, a cross-modal differentiable sorting loss, and our proposed consistency constraint loss. A standard cross-entropy function is employed as the identity loss to ensure the discriminativeness of features.

Additionally, we follow [36] and employ a cross-modal differentiable sorting loss for metric learning. Unlike the traditional triplet loss, this loss treats each training batch as a mini retrieval task. Specifically, each sample in the batch serves as a query, while samples from the other modality form the gallery. We first compute the cosine distances between the query and all gallery samples to form a distance vector $D_{i}$. A differentiable ranking operator ϕ(·), is then applied to this vector to generate the predicted ranking list, $R_{i}=\phi \left(D_{i}\right)=\;\left[r_{1},\ldots ,r_{n}\right]$. Concurrently, we define a target ranking list ${\hat{R}}_{i}$ based on the ground-truth identity labels. This target list assigns all positive samples at the first rank and all negative samples at the last:

(10) $${\hat{r}}_{j}=\left\{\begin{array}{cc} 1, & \mathrm{if}\;\mathrm{id}\left(g_{j}\right)=\mathrm{id}\left(q_{i}\right)\\ n, & \mathrm{otherwise} \end{array}\right.,$$

where id(·) denotes the identity label of a sample.

The cross-modal differentiable sorting loss is defined as the average Spearman’s footrule distance between the predicted and target rankings across the batch:

(11) $$L_{cmds}=\frac{1}{B\cdot n}\sum_{i=1}^{B}\sum_{j=1}^{n}\left|r_{ij}-{\hat{r}}_{ij}\right|,$$

where *B* is the batch size, *n* is the gallery size, and $r_{ij}$ and ${\hat{r}}_{ij}$ are the predicted and target ranks, respectively, for the *j*-th gallery sample in the *i*-th query.

Therefore, the overall training loss becomes:

(12) $$L_{total}=L_{id}+L_{cmds}+\lambda L_{cons},$$

where λ is hyperparameter that controls the relative importance of each loss component.

## 4. Experiments

In this section, we first introduce the sketch datasets and the evaluation protocols, followed by a description of the experimental settings. Then we present comparisons with state-of-the-art methods on the Pku-Sketch [22] and Market-Sketch-1K [30] datasets. Next, we describe in detail the experimental structure of our model, component analysis, ablation experiments and visualization results.

### 4.1. Datasets and Evaluation Protocols

We evaluate our model on two publicly available sketch datasets, including PKU-Sketch [22] and Market-Sketch-1K [30] dataset. A summary of the descriptive information for these datasets is provided in Table 1.

PKU-Sketch is the first sketch dataset for person Re-ID and consists of 200 pedestrians. Each pedestrian has two photographs and one sketch image. The photographs are captured by cross-view cameras from outdoors, and all sketch images are drawn by five professional artists. Following the strategy proposed by Pang et al. [22], we randomly selected 150 pedestrians with 300 photographs and 150 sketches from each painting style for the training set and the remaining 50 identities to the testing set.

**Figure 4:**
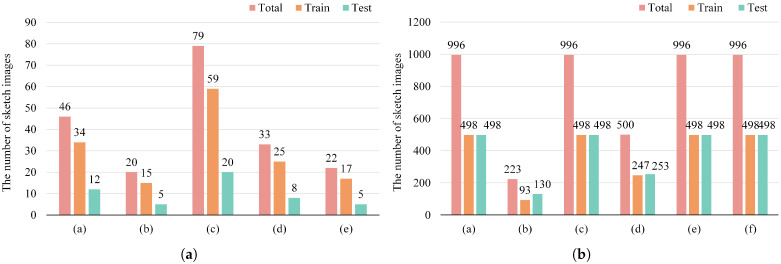
(**a**) The number of sketch images drawn by different professional painters and the division of training and test sets in the PKU Sketch dataset. (**b**) The number of sketch images drawn by different professional painters and the division of training and test sets in the Market-Sketch-1K dataset.

**Figure 5:**
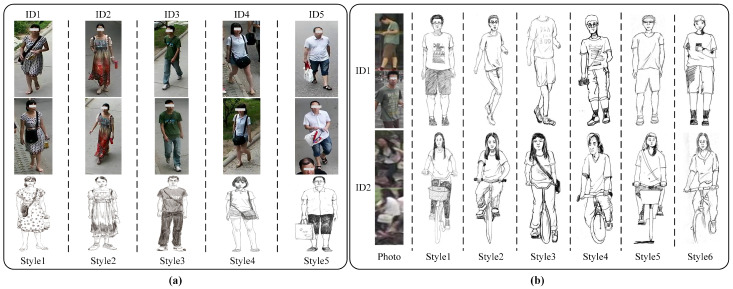
(**a**) Samples with different cameras and painting style from PKU-Sketch dataset. (**b**) Samples with different cameras and painting style from Market-Sketch-1K dataset.

Market-Sketch-1K is a large-scale dataset derived from the widely-used Market-1501 [5] dataset. The sketch images in this dataset were produced by six professional artists, each providing their own interpretation of the source photographs, resulting in a rich variety of styles. The training set contains 498 identities, with 12,936 photos and 2332 sketches, while the testing set consists of another 498 identities, with 19,732 photos and 2375 sketches. Following the experimental protocol in [30], our method is evaluated under two scenarios: single-query and multi-query. Figure 4 summarizes the number of different painting styles in the PKU-Sketch and Market-Sketch-1K dataset and shows the specific distribution of training and testing sets. Figure 5 presents representative image samples from PKU-Sketch and Market-Sketch-1K dataset.

We followed the standard ReID evaluation metrics and use cumulative matching characteristic (CMC) [37], mean average precision (mAP) [5] and the the mean inverse negative penalty (mINP) [26] to assess the performance of our proposed model. Specifically, when presented with a query sketch, all images are ranked according to their similarity to the given sketch. The CMC shows the proportion of correct matches in Rank-k, a higher position of the correct matching images in the ranking indicates superior model precision, and the mAP is computed by averaging the retrieval precision across all categories. All reported results represent the mean values obtained from 10 evaluations on the test set.

### 4.2. Implementation Details

Our model is implemented using the PyTorch framework, with a single NVIDIA GeForce RTX 4090 GPU. We used a ResNet50 [38] network pre-trained on ImageNet [39] as the backbone to extract features, and we insert our proposed MDP modules at 5 layers (L=5). The input images are resized to 384×192. We utilized a standard data augmentation pipeline, including random horizontal flipping, random cropping, and Random Erasing. For each training batch, we randomly sampled 4 identities and 8 images per identity. To ensure modality balance, for each sampled identity, we specifically sample 4 photos and 4 sketches. If an identity has fewer than 4 samples for a given modality, random oversampling with replacement is applied to meet the required count, ensuring each identity within a batch simultaneously includes samples from both modalities. To optimize the model’s performance, we employed the Adam optimizer. During the training phase, the initial learning rate is set to 3.5 $\times \;10^{-4}$. The learning rate was reduced by a factor of 10 at epochs 80 and 120. The total number of training epochs is set to 150. The optimal weight λ of the cross-layer decoupling consistency loss was found to be correlated with the intrinsic characteristics of each dataset. Specifically, λ is set to 0.2 for the PKU-Sketch dataset and to 0.5 for the more challenging Market-Sketch-1K dataset. A detailed sensitivity analysis of λ is presented in Section 4.5.

### 4.3. Comparison with State-of-the-Arts

We first compared our proposed method with the state-of-the-art cross-modality retrieval models on the Pku-Sketch dataset. The quantitative results are presented in Table 2, Table 2 shows the results, with the best results shown in bold. These methods were classified into several categories: traditional methods based on hand-crafted edge maps (e.g., Triple SN [40], GN Siamese [41]), metric learning approaches by learning shared embeddings (e.g., LDMI [23], CDA [24], IHDA [27]), and generative models (e.g., CD-AFL [22], SketchTrans [7]). Our method achieved a Rank-1 accuracy of 88.37% and a mAP of 84.2%, outperforming most of the others and demonstrating competitive results. Early methods such as Triple-SN [40] employed manually extracted edge maps and hand-drawn sketches for feature extraction, yielding unsatisfactory performance in this challenging scenario. Approaches such as CD-AFL [22], which pioneered the use of GANs to filter out domain-specific noise, and attention-based models like LDMI [23] and CDA, primarily focus on learning modality-invariant features. Our method demonstrates a substantial performance lead over these approaches, indicating the superiority of our feature decoupling strategy. CD-AFL [22] is the first method specifically designed for sketch Re-ID task, utilizing cross-modal generative adversarial networks to learn modality-invariant representations. Methods such as LDMI [23] and CDA [24] focus on learning modality-invariant features through spatial attention or inter-domain attention mechanisms. Compared to these methodologies, our approach demonstrates substantial improvements, indicating the superiority of our feature decoupling strategy. When compared to methods that leverage additional attribute annotations to bridge the photo-sketch gap (e.g., IHDA [27], beyondDG), our approach achieves superior performance without requiring such auxiliary information. Furthermore, when compared to methods that leverage additional attribute annotations to bridge the photo-sketch gap (e.g., IHDA [27], subjectivity [30]), our approach achieves superior performance without requiring such auxiliary information. Moreover, our method also surpasses methods such as CSIG [29] and MSIF [6], which augment the training data pool via multi-spectrum image fusion. However, such methods lack a reliable mechanism to narrow the modality gap at the feature level. Besides, compared to feature disentanglement like SketchTrans [7] and SketchTrans+ [7], which employed an asymmetric decoupling learning on photo features, our method adopts a symmetric network to simultaneously conduct multi-level disentanglement on both photographs and sketches. This symmetric design ensures that the model learns stable and coherent disentangled features, effectively enhancing the discriminative power of learned modality-invariant representations and thereby improving overall cross-modal retrieval performance.

The results across all test modes of our experiments on the Market-Sketch-1K dataset are shown in Table 3. In contrast to PKU-Sketch dataset, the sketches in Market-Sketch-1K exhibit greater abstraction and sparser detail information, substantially increasing the difficulty of the cross-modal Re-ID. As shown in Table 3, our method demonstrates a clear superiority over all state-of-the-art (SOTA) competitors in both evaluation settings. In the single-query scenario, the proposed method achieves 19.96% Rank-1 and 22.33% mAP accuracy, surpassing the best performing baseline by 1.86% and 2.72%, respectively. For multi-query evaluation, our approach attains 31.53% Rank-1 and 31.36% mAP accuracy. Our method outperforms mainstream cross-modal Re-ID approaches(e.g., DDAG [43], CM-NAS [44]) or standard shared-embedding learning (e.g., CAJ [45], DART [46]). Approaches such as DCLNet [47] focused on fine-grained pixel alignment, are inherently handicapped by the sparse nature of sketches, which makes reliable pixel-to-pixel correspondence difficult to establish. DSCNet [12] reduces feature distribution disparity by constraining inter-channel semantic and inter-modal semantic consistency. However, this method fails to adequately learn modality-invariant structural information. Our approach focuses on disentangling modality-invariant structural cues and modality-specific appearance cues and ensuring semantic coherence across different levels, achieves superior performance in both Rank-1 accuracy and mAP. Compared with the data augmentation techniques in CAJ [45], DEEN [48] and MCJA [49], our method considers the distinct image characteristics of sketches and photos, demonstrating substantial superiority over these approaches. Furthermore, when compared to generative methods such as MMN [50] and SketchTrans+ [7], which seek to bridge the modality gap by synthesizing intermediate images, our approach is more direct and efficient. It achieves superior results without requiring an auxiliary generation network, thus avoiding additional computational overhead and the risk of introducing noise that can disrupt the matching process. Besides, compared with the Subjectivity [30] method, our approach requires no extra attribute annotations while demonstrating significant performance advantages in both single-query and multi-query scenarios.

### 4.4. Ablation Study

To evaluate the contributions of each component within our proposed network, we conducted a series of ablation studies on the PKU-Sketch and Market-Sketch-1K dataset. We removed the proposed multi-channel color augmentation module, the modality-disentangled prototype module and cross-layer decoupling consistency loss from the model to create a baseline, where only the final id loss and triple loss were used for training. Herein, we use the abbreviations ‘MCCA’ for the multi-channel color augmentation module, ‘MDP’ represents the modality-disentangled prototype module, and ‘$L_{cons}$’ for the cross-layer decoupling consistency loss. The detailed experimental results are summarized in Table 4.

The results in Table 4 demonstrate that our proposed MCCA module, MDP module and the cross-layer decoupling consistency loss all significantly enhance the model’s capacity for learning modality-invariant features. Initially, by incorporating the MCCA module into the Baseline, the model’s Rank-1 and mAP increased by 9.24% and 11.55%, respectively. This indicates that MCCA module effectively mitigates the impact of color asymmetry between sketches and photos, guiding the model to learn more robust, color-agnostic discriminative information. Notably, a comparison between rows 2 and 3 of Table 4 reveals that our symmetrical application of MCCA—augmenting both photo and sketch modalities further performance gains over that targets only photos. This validates our design choice. The MDP module is designed to disentangle features into structural and appearance prototypes. When combined with cross-layer disentanglement consistency loss, this combination compelled the network to prioritize structural information while suppressing modality-specific appearance-related interference. It can be observed that after incorporating the MDP module, there were significant improvements in rank-1 accuracy and mAP on the PKU-Sketch dataset over the baseline, with increases of 12.83% and 10.98%, respectively. When all components are integrated, our full model achieves the optimal performance, confirming the synergistic contribution of each module.

To further validate the effectiveness of our proposed multi-channel color augmentation module, we evaluated MCCA’s three constituent strategies by integrating each one individually into the baseline model. The results are presented in Table 5. The strategies that under investigation are: Grayscale Conversion(GC), Intensity Perturbation(IP), and Cross-channel Mixing(CCM). The experimental results demonstrate that each individual color augmentation strategy effectively enhances model performance. Compared with the baseline, our GC, IP, and CCM each bring a +10.38%, +6.96%, +6.31% rank-1 accuracy and a +6.19%, +3.6%, +4.38% mAP improvement, respectively. This demonstrated that all three strategies were effective at generating diverse training samples, which in turn compels the model to learn features that are robust to the color information asymmetry inherent in the sketch-photo recognition.

We also report Rank-1 accuracy and mAP on the PKU-Sketch and Market-Sketch-1K datasets, grouped by artist drawing style. From Table 6, we observe that for certain fine-grained drawing styles (e.g., Style_2 in PKU-Sketch and Style_1 in Market-Sketch-1K), our model achieves higher Rank-1 and mAP than the overall average results reported in Table 2 and Table 3. Considering Figure 5 and Table 1, we believe this is because such styles preserve more identity-related structural and contour information, enabling the MDP module to extract more stable structural prototypes. We also note lower performance in certain styles, such as Style_5 in Market-Sketch-1K. We attribute this to the highly abstract or oversimplified nature of these sketches, which fail to preserve sufficient discriminative features of the person, leading to significant information loss and posing greater challenges for sketch–photo matching. Nevertheless, our method demonstrates robustness in handling such extreme modality differences. By emphasizing modality-invariant structural prototypes, our model still achieved effective matching even under these information-sparse conditions.

### 4.5. Parameter Analysis

**Figure 6:**
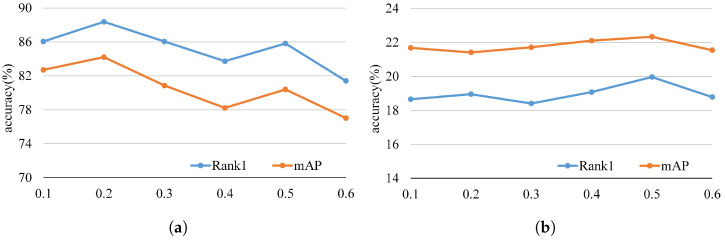
(**a**) Evaluation of the effect of different values of λ on Pku-Sketch dataset. (**b**) Evaluation of the effect of different values of λ on Market-Sketch-1K dataset.

We further examine the influence of the weight hyperparameter λ of the cross-layer disentanglement consistency constraint on the overall model performance. As illustrated in Figure 6, we present Rank-1 accuracy and mAP results on both the PKU-Sketch and Market-Sketch-1K datasets, with λ varied from 0.1 to 0.6 in increments of 0.1. From Figure 6a,b, we observed that the performance curves on both datasets follow a trend of initial improvement followed by gradual degradation. This demonstrated that cross-layer disentanglement consistency constraint effectively guided model training and enhances performance. Insufficient λ values fail to provide adequate decoupling constraints, while overly large values imposes excessive regularization that degrades learning. Furthermore, the optimal λ value differs between the two datasets. Specifically, the model achieved its best performance on PKU-Sketch dataset when λ equals 0.2, while the optimum for the more challenging Market-Sketch-1K dataset is reached at λ equals 0.5. This discrepancy suggests that due to its larger scale and greater modality variation, the Market-Sketch-1K dataset requires stronger decoupling-consistency regularization to ensure a stable disentanglement strategy.

### 4.6. Visualization of Results

**Figure 7:**
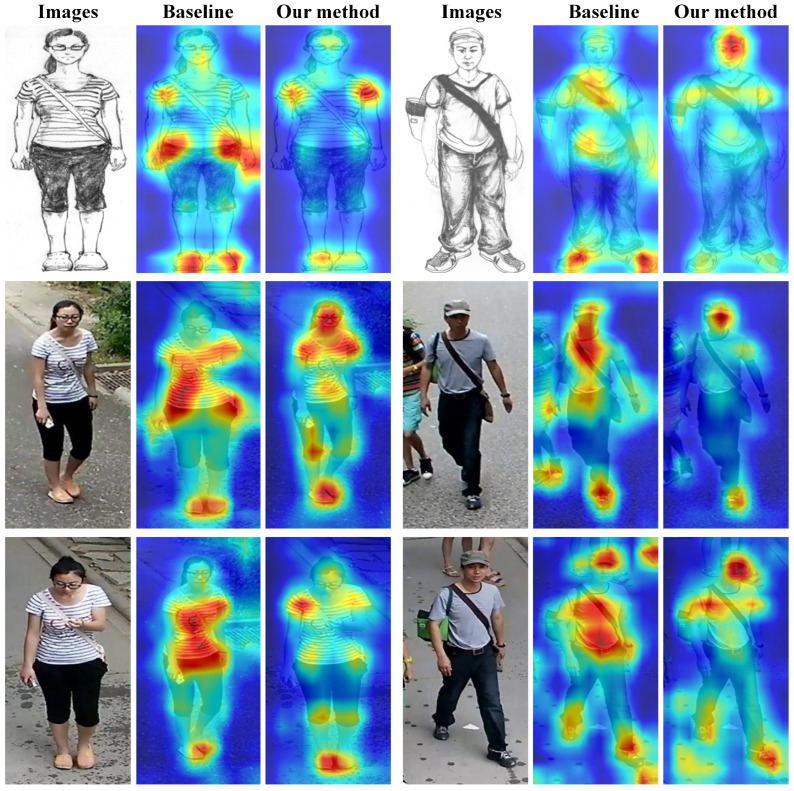
Visualization of feature heatmaps on the PKU-Sketch test set. The first row shows the heatmap of the sketch image, while the second and third rows display the heatmaps of photo images corresponding to the same identity.

To further elucidate the underlying mechanism of our proposed method, we employ Grad-CAM [51] to visualize the attention maps of both the baseline model and our proposed model. As presented in Figure 7, the visualizations revealed that the baseline model exhibits a dispersed and inconsistent attentional focus. Its activations tend to concentrate on salient yet modality-specific regions. For example, for the first person the sketch attention map emphasized the limb regions, whereas the photo attention map predominantly focused on the striped texture of the T-shirt. In contrast, our method consistently localizes modality-invariant structural cues (e.g., body contours and limbs) across both modalities and shows reduced responses to transient appearance details. These observations indicated that the proposed method effectively guided the network to disentangle modality-specific appearance cues from modality-invariant structural representations, thereby enhancing robustness against significant cross-modal variations.

**Figure 8:**
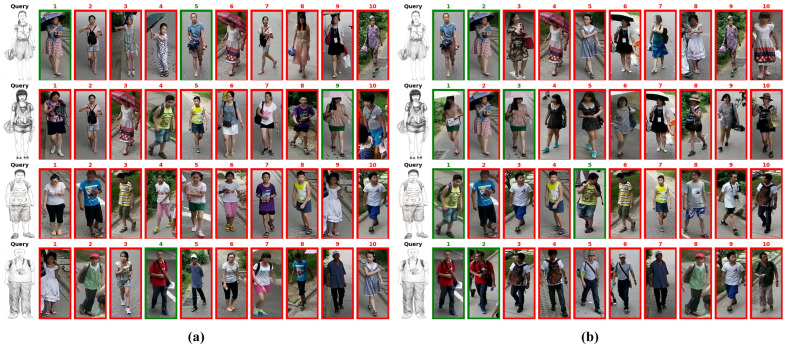
Visualization of retrieval results on the PKU-Sketch dataset. (**a**) presents the baseline results, and (**b**) shows the results obtained by our method. The correct retrieval results are in green boxes.

**Figure 9:**
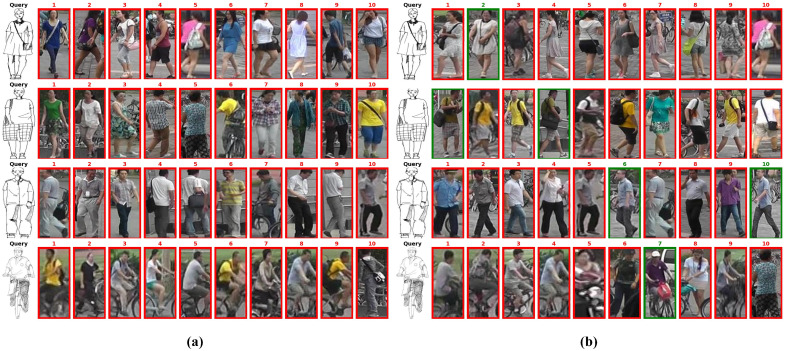
Visualization of retrieval results on the Market-Sketch-1K dataset. (**a**) presents the baseline results, and (**b**) shows the results obtained by our method.The correct retrieval results are in green boxes.

Figure 8 and Figure 9 presented qualitative retrieval results on the test sets for both datasets. Based on these results, our method demonstrates the following advantages: (1) The multi-channel color augmentation strategy enables our model to learn modality-invariant features beyond color information, as illustrated by examples from the Market-Sketch-1K dataset dataset in Figure 9. (2) Our method significantly reduces intra-class distances on both the fine-grained Pku-Skecth dataset and the more challenging Market-Sketch-1K dataset, demonstrating that modality-disentangled prototype learning can obtain cross-modal representations more effectively. (3) The proposed method effectively focuses on modality-independent contour and structural information by combining multi-channel color augmentation with modality-disentangled prototype learning. Specifically, in both Pku-Skecth and Market-Sketch-1K dataset, certain local regions of pedestrian samples were highly similar and prone to mismatch. Compared to baseline models, our approach successfully extracts modality-invariant features, thereby enhancing robustness against interfering samples and cross-modal variations.

## 5. Conclusions

This paper concentrates on the sketch-photo re-identification task. The information heterogeneity across two modalities, particularly the asymmetry of color information, poses significant challenges for cross-modal learning. To tackle this, we proposed a novel hierarchical disentanglement learning method based on multi-channel color enhancement to learn representative modality-invariant embeddings at both data and feature levels. First, at the data level, we introduced a multi-channel color enhancement module that applies diverse color transformations to input images, encouraging the network to learn deeper deeper shared features beyond color information. Second, at the feature level, we designed a modality disentangled prototype module that explicitly disentangled sketch and photo features into modality-invariant structural prototypes and modality-specific appearance prototypes. Furthermore, to ensure semantic coherence and stability of these prototypes across different network layers, we introduce a cross-layer decoupling consistency constraint. This constraint guides the network to learn more robust cross-modal features, ultimately enhancing the model’s recognition performance. Extensive experiments on two public sketch-based person re-identification datasets demonstrate the superiority of our proposed method. Given the limited number of publicly available Sketch-ReID datasets, further validating our method on larger, more diverse sketch collections is an important direction for future work. In the future, We plan to improve and verify the model’s cross-domain robustness through additional data collection, synthetic data augmentation, and unsupervised domain adaptation.

## Figures and Tables

**Table 1:** Descriptive information of sketch datasets.

Datasets	IDs	Photos	Sketches	Cameras	Styles
PKU-Sketch	200	400	200	2	5
Market-Sketch-1K	996	32,668	4763	6	6

**Table 2:** Comparison to state-of-the-art methods on PKU-Sketch dataset. Rank (R) at k accuracy and mAP (%) are reported.

Method	Source	Rank1	Rank5	Rank10	Rank20 *	mAP *
Triple SN [40]	CVPR’16	9.00	26.80	42.20	65.20	-
GN Siamese [41]	TOG’16	28.90	54.00	62.40	78.20	-
CD-AFL [22]	MM’18	34.00	56.30	72.50	84.70	-
LDMI [23]	Neuro’20	49.00	70.40	80.20	92.00	-
IHDA [27]	TMM’20	85.00	94.80	98.00	100	-
CDA [24]	TIFS’22	60.80	80.60	88.80	95.00	-
CSIG [29]	IFTC’22	77.60	93.00	97.00	98.80	-
CCSC [42]	MM’22	86.00	98.00	100	-	83.70
SketchTrans [7]	MM’22	84.60	94.80	98.20	99.80	
MSIF [6]	IJMLC’24	87.00	96.80	98.70	98.82	**91.12**
SketchTrans+ [7]	TPAMI’24	85.80	96.00	99.00	99.30	-
Ours	-	**88.37**	**95.35**	**99.80**	**100**	84.20

* ‘-’ indicates the metric was not reported in the original paper.

**Table 3:** Comparison to state-of-the-art methods on Market-Sketch-1K dataset. Rank (R) at k accuracy and mAP (%) are reported.

Method	Source	Query	Rank1	Rank5	Rank10	Rank20 *	mAP
DDAG [43]	ECCV’20	S	11.22	25.40	35.02	-	12.13
CM-NAS [44]	ICCV’21	S	0.70	2.00	3.90	-	0.82
CAJ [45]	ICCV’21	S	1.48	3.97	7.34	-	2.38
MMN [50]	MM’21	S	9.32	21.98	29.58	-	10.41
DART [46]	CVPR’22	S	6.58	16.75	23.42	-	7.77
DCLNet [47]	MM’22	S	12.24	29.20	39.58	-	13.45
DSCNet [12]	TIFS’22	S	13.84	30.55	40.34	-	14.73
DEEN [48]	CVPR’23	S	12.11	25.44	30.94	-	12.62
Subjectivity [30]	MM’23	S	18.10	38.95	50.75	-	19.61
		M	24.70	50.40	63.45	-	24.45
MCJA [49]	TCSVT’24	S	14.51	33.67	44.73	58.19	15.86
		M	27.31	52.01	65.66	76.51	26.39
Ours	-	S	**19.96**	**40.08**	**52.24**	**66.08**	**22.33**
		M	**31.53**	**51.2**	**62.65**	**75.9**	**31.36**

* ‘-’ indicates the metric was not reported in the original paper.

**Table 4:** Ablation study. Performance comparison of each components in our method on Pku-Sketch dataset and Market-Sketch-1K dataset with rank-1,(%), mAP(%) and mINP(%).

Method	Pku-Sketch			Market-Sketch-1K		
	**Rank-1**	**mAP**	**mINP**	**Rank-1**	**mAP**	**mINP**
Baseline	65.43	64.03	53.16	6.41	9.09	4.15
Baseline + MCCA(only RGB)	74.67	75.58	62.71	11.52	13.18	6.40
Baseline + MCCA	82.22	79.50	64.18	17.69	18.08	8.79
Baseline + MDP	78.26	75.01	63.17	11.05	13.14	6.34
Baseline + MDP + Lcons	80.89	76.54	68.05	11.43	13.33	6.63
Our Method	**88.37**	**84.20**	**77.34**	**19.96**	**22.33**	**15.50**

**Table 5:** Comparative the performance of individual augmentation strategies within the MCCA module on the PKU-Sketch dataset.

Method	Rank-1	Rank-5	Rank-10	Rank-20	mAP	mINP
Baseline	65.43	88.04	93.91	99.78	64.03	53.16
Baseline + GC	75.87	93.70	98.04	100	70.22	58.11
Baseline + IP	72.39	96.52	99.20	100	67.63	54.78
Baseline + CCM	71.74	90.43	98.26	99.57	68.41	57.24
Our Method	**88.37**	**95.35**	**99.80**	**100**	**84.20**	**77.34**

**Table 6:** Single-style query evaluation. On the PKU-Sketch and Market-Sketch-1K datasets, we report results using queries of a single style.

Sketch Style	Pku-Sketch		Market-Sketch-1K	
	**Rank-1**	**mAP**	**Rank-1**	**mAP**
Style_1	82.14	69.10	30.51	27.88
Style_2	100.00	93.67	12.50	15.96
Style_3	94.96	89.23	21.21	23.02
Style_4	77.50	57.91	12.75	17.75
Style_5	82.33	82.99	11.50	13.75
Style_6	-	-	22.04	21.33

## Data Availability

The original contributions presented in this study are included in the article. Further inquiries can be directed to the corresponding author(s).
